# Personal initiative and work environment as predictors of job satisfaction among nurses: cross-sectional study

**DOI:** 10.1186/s12912-021-00615-1

**Published:** 2021-06-06

**Authors:** Ilya Kagan, Tova Hendel, Bella Savitsky

**Affiliations:** 1 Nursing Department, Sackler Faculty of Medicine, School of Health Professions, Tel Aviv University, Ramat Aviv, Israel; 2 Department of Nursing, School of Health Sciences, Ashkelon Academic College, Yitshak Ben Zvi 12, Ashkelon, Israel

**Keywords:** Job satisfaction, Personal initiative, Work environment, Nurses, Hospitals

## Abstract

**Background:**

Job satisfaction contributes to better work outcomes and productivity, and reduces nurses’ absenteeism and turnover. The contribution of personal initiative to the interaction between these variables needs additional examination. This study aimed to examine the relationships between personal initiative, work environment, and job satisfaction among nurses.

**Methods:**

This was a cross-sectional study. The convenience sample consisted of 1040 nurses working in hospitals across the country. Data were collected by a structured self-administered questionnaire measuring: (a) personal initiative, (b) nursing work environment, (c) job satisfaction.

**Results:**

Personal initiative and work environment scores, together with demographic and occupational characteristics that univariate analysis showed to be significantly associated with job satisfaction, were included in a logistic regression model to predict job satisfaction. The results of multivariable analysis indicated that female gender, working in emergency room (ER) and pediatric wards, a higher personal initiative, and positive perception of work environment, were significantly associated with higher job satisfaction. Work in the ER and pediatric area of practice was significantly associated with five-fold (OR = 4.97; 95% CI 1.52–16.25) and three-fold higher odds (OR = 2.85; 95% CI 1.17–6.91) for high and very high job satisfaction in comparison with work in oncology. The model explained 32% of the variance in job satisfaction**.**

**Conclusions:**

The findings demonstrate that high personal initiative together with positive perceptions of the nursing work environment, contributed significantly to the explanation of job satisfaction. There is a need to invest more efforts in strengthening the organizational climate stimulating initiative behavior and encouraging nurses to be active, share knowledge, and promote innovation.

## Background

Since people spend a significant part of their life at work, it is not surprisingly that studies consistently show that individuals, who are more satisfied with their job, are not only happier but also healthier. The risk of depression, as well as anxiety and stress are all lower among satisfied workers [1], and job satisfaction has positive effects on health, happiness, subjective well-being, and self-esteem [2]. A meta-analysis of almost 500 studies demonstrated strong relationships between job satisfaction and both mental and physical health [3]. In terms of organizational benefit, job satisfaction contributes to better work performance and outcomes, improves work productivity, and reduces absenteeism and turnover [4–6]. Nursing studies have generally concluded that a significant number of resignations are due to job dissatisfaction [7].

A number of theories have attempted to explain the phenomenon of job satisfaction. Frederick Herzberg’s two-factor theory [8] claims that two types of factors influence job satisfaction. Hygiene factors (extrinsic or maintenance factors) are represented by job security, salary, fringe benefits, work conditions, etc. The second type of factors are the motivators, such as high skill requiring work, recognition for better performance, responsibility, autonomy, meaningfulness, involvement in decision making, organizational commitment, etc.. These factors provide positive satisfaction, arising from the intrinsic conditions of the job itself, such as recognition, achievement, and personal growth. The results of a study that investigated this issue demonstrated that the presence of extrinsic factors merely succeeds in preventing job dissatisfaction [9]. Only the addition of intrinsic factors could cultivate employees’ inner growth and development and promote higher productivity, improved performance, and higher job satisfaction.

Job satisfaction of nurses can be influenced by a variety of extrinsic and intrinsic factors. Work environment is a well-known predictor of nurses’ job satisfaction, as an extrinsic factor, while personal initiative can play a role as an intrapersonal (intrinsic) characteristic [10]. Personal initiative is a behavior defined as self-starting and proactive that overcomes barriers to achieve a goal [11]. This is a key characteristic for innovation as employees who are creative and innovative must also be proactive [12, 13].

Innovation is an important component of a healthy work environment. Healthcare innovation can be defined as “the introduction of a new concept, idea, service, process, or product aimed at improving treatment, diagnosis, education, outreach, prevention, and research, and with the long term goals of improving quality, safety, outcomes, efficiency and costs” [14]. The recently updated American Nurse Association scope and standards of practice calls for all nurses to lead, be involved in policy practice, and promote innovation [15].

Health care systems are currently undergoing a rapid advance. This is characterized by the introduction of new technology and approaches for enhancing life expectancy, quality of life, and diagnostic and treatment options, while still taking efficiency and cost effectiveness of the healthcare system into account. Innovation has become a crucial element of the modern work environment, and nurses play a very central role in this progress [16]. Personal initiative is a behavioral phenomenon that involves developing a fuller set of goals that go beyond the formal requirements of the job, and being pro-active [17]. The extent of the initiative behavior displayed depends on nurses` characteristics and their work environment.

Personal initiative has been positively associated with innovative behavior [18]. Personal initiative is affected by the work environment climate [19], and in turn positively influences the working milieu. To the best of our knowledge, this is the first study in Israel to address the role of personal initiative in the context of work environment and nurses’ job satisfaction.

### Aim

This study was designed to examine the relationships between personal initiative, work environment characteristics, and job satisfaction among Israeli nurses working in general hospitals.

## Methods

### Study design and sample

The data in this cross-sectional design study were collected using a structured self-administered questionnaire. The convenience sample consisted of 1040 nurses working in hospitals across Israel. The collection of data began after receiving the approval from the nursing management in the department and the clinical division. Only departments that provided an approval, participated in the study. A total of two or three departments in each medical organization were selected and their personnel were invited to participate in the study. The questionnaires were distributed by senior nurses working in the wards, together with a letter explaining the aim of the study and guaranteeing respondents’ anonymity and confidentiality. Those who gave their verbal consent to participate, were asked to complete the questionnaire. The compliance rate ranged between 75 and 88% in different settings.

### Study variables

Data were collected by a structured self-administered questionnaire comprising four parts, measuring: (a) personal initiative, (b) nursing work environment, (c) job satisfaction, and (d) respondent’s demographic data.

*The personal initiative* variable was measured by a 14-item scale developed and validated by Frese et al. [20]. The tool comprised two sections: a) *Self-reported initiative* (7 items) focused on activity and innovation in dealing with unexpected difficulties, problem-solving, and achieving goals, and b) *Passivity* (7 items) representing inactiveness in planning career, adjustment to changing environment, and responding to challenges at work. Participants were asked to rank each statement on a 6-point scale from 1 (absolutely disagree) to 6 (totally agree). The final score was represented by the mean value, with higher scores indicating a higher personal initiative. We used a Hebrew version of the tool [19]. In our previous study [19], the Cronbach’s alpha score was 0.78, and in the current analysis was 0.77. The personal initiative score displayed a normal distribution and was treated as a continuous variable.

*Nursing work environment* variable was measured by 14 items from the Revised Nursing Work Index (NWI-R) [21] that assess nurse autonomy, control over nursing practice, nurse-physician relations, and team working. Again, we used the Hebrew version of the tool [19]. The items included in the final version of this section were chosen and validated by five senior nurses working in a general hospital. For this purpose, they were asked to identify items that represent a work environment and have a possible impact on the initiative behavior of staff nurses. All the selected items were chosen by a full consensus. Respondents were asked to score their degree of agreement on a scale from 1 (strongly disagree) to 5 (strongly agree). One item was recoded. The overall score of this part was represented by the mean. In our previous study, the Cronbach’s alpha score for this instrument was 0.85 [19], in the current study, it was 0.90. The nursing environment score displayed a normal distribution and was treated as a continuous variable.

*Job satisfaction* variable was measured with a 10-item tool [22]. This part of the questionnaire is focused on the self-fulfillment (internal) aspects of working - the achievement of personal and professional goals, belonging, social recognition, and self-realization at work. Respondents were asked to rank the extent of their agreement with statements on a scale of 1 (very low) to 5 (very high). The job satisfaction score was represented by the mean. The internal reliability score in earlier studies was 0.91–0.93 [22, 23]. In the current study, Cronbach’s alpha was 0.90. The mean satisfaction score for all participants was calculated. The distribution of this score was not normal, and this variable was therefore further categorized into two categories: 1) high and very high job satisfaction (a mean score of 4.0+), and 2) low to medium job satisfaction (mean score < 3.9).

The *respondents’ demographic data* were collected using the tool from our previous study [19] measuring as follows: *Age* was used as a continuous variable and as a dichotomy variable with the median as the cut-off point (40 years); *Sex:* Female and Male. *Country of birth* was used as a categorical variable with categories: Israel; Former Soviet Union (FSU); Other. *Level of professional education* had three categories: RN – registered nurse; RN BA – RN with Baccalaureate degree; RN MA – registered nurse with Master’s degree. *Seniority* (years of professional experience) was used as a continuous variable and as a dichotomy variable with the median as the cut-off point (14 years). *Type of hospital* was used as categorical variables with categories: General; Pediatric; Psychiatric. *Form of Employment* was used as a categorical variable with categories: 100%; 75–90%; 25–66%. *Area of practice* was used as a categorical variable with categories: Surgery; Psychiatry/Rehabilitation; Intensive Care Units (ICU); Pediatric/Newborn/Premature; Internal Medicine/Geriatric; Emergency Room (ED); Oncology; Obstetrics & Gynecology; Operating Rooms (OR); Other.

### Statistical data analysis

Student’s *t*-test and ANOVA were used to investigate the presence of an association between demographic/occupational characteristics, personal initiative, and work environment variables, with *χ*^*2*^ for the association between demographic/occupational variables and job satisfaction. Demographic and occupational characteristics that univariate analysis demonstrated to be significantly associated with job satisfaction, were included (in addition to personal initiative and work environment scores) in a multivariable logistic regression model for prediction of high and very high job satisfaction. Before including independent variables in multivariable analysis, the correlations between the variables were verified with Kendall’s Tau coefficient. Most of the correlations were weak, with the strongest reaching a Kendall’s Tau coefficient of 0.175. The level of significance employed throughout was 0.05. Data were analyzed using SPSS 25.0 (IBM, USA).

### Ethics approval and consent to participate

Approval for this study was obtained from the Ethics Committee (IRB) of Tel Aviv University (not numbered). The need to sign an informed consent form was waived but verbal consent was obtained from the participants prior to inclusion in this study.

## Results

### Descriptive statistics

The demographic and occupational characteristics are shown in Table 1. Half the participants were older than 40, 75% were female and had longer professional experience. Most members of the sample (58%) were Israel-born. Only 20% of the nurses were RNs without an academic degree, while 60% had a bachelor’s degree (RN BA), and 20% had a master’s degree (RN MA). Most of the participants worked full time, with a higher proportion of men working full time than the women (91% vs. 64%).

**Table 1:** Study population demographic and occupational characteristics by sex

Demographic and occupational characteristics		Male *n* = 247 (24%)	Female *n* = 779 (75%)	Total* n = 1040
Age**, years (mean, SD)		38.8 (9.5)	40.6 (9.7)	40.2 (9.7)
Country of birth** (%)	Israel	70.8	54.1	58.1
	FSU	25.9	39.3	36.1
	Other	3.3	6.6	5.8
Seniority in the profession*, years (mean, SD)		12.4 (9.3)	15.1 (10.3)	14.5 (10.1)
**Education**	RN	17.8	20.3	19.7
	RN BA	62.3	58.7	59.6
	RN MA	19.8	21.1	20.8
**Form of Employment**	100%	90.6	64.1	70.5
	75–90%	7.8	28.5	23.5
	25–66%	1.6	7.4	6.0
**Type of Hospital**	General	52.2	58.7	57.1
	Pediatric	7.7	18.5	15.9
	Psychiatric	40.1	22.8	27.0
**Area of practice** (%)	Surgery	22.7	24.6	24.1
	Psychiatry/Rehabilitation	31.2	21.0	23.5
	Intensive Care Units	14.2	16.5	15.9
	Pediatric/Newborn/Premature	2.4	13.4	10.8
	Internal Medicine/Geriatrics	7.7	11.1	10.3
	Emergency Room	4.0	3.7	3.8
	Oncology	5.7	2.7	3.4
	Obstetrics & Gynecology	0.8	3.0	2.4
	Operating room	3.6	1.5	2.1
	Other	7.7	2.4	3.7

* Among 14 participants sex is missing

** The percentage of the missing values = 1%

Most members of the sample (57%) worked in general hospitals, although relatively more men than women worked in a psychiatric hospital (40% vs. 23%). Surgery and psychiatry/rehabilitation were the most frequent areas of practice, with almost 25% of participants working in one or other of these areas.

### Personal initiative, work environment, and job satisfaction

The associations of demographical and occupational characteristics with Personal Initiative, Work Environment and Job Satisfaction Scores, by univariate analysis, are shown in Table 2. A higher Personal Initiative Score was significantly associated with younger age, Israeli-origin, MA academic degree, full-time employment, and working in the operating room. A higher Work Environment Satisfaction Score was significantly associated with higher seniority in the profession, MA academic degree, working in a psychiatric hospital, full-time job, and surgery, psychiatry and rehabilitation, or operation room, as the area of practice. Higher Job Satisfaction was observed among women (73% of them reported high and very high job satisfaction vs. 64% of men), and nurses with an MA academic degree (78% of RN MA reported high and very high job satisfaction vs. 67–70% in the RN and RN BA groups, respectively). The lowest proportion of nurses who reported high and very high job satisfaction was observed in oncology (47%) and internal/geriatric and OR areas of practice (67%) vs. other areas (72–83%). The highest proportion of nurses who reported high/very high job satisfaction, worked in the ER (83%). Age, professional seniority, and format of employment were not associated with job satisfaction.

**Table 2:** Association between socio-demographic characteristics and personal initiative, work environment, and job satisfaction

Demographic and occupational characteristics		Personal Initiative Score	Work Environment Score	Job Satisfaction Score
		Mean (SD)		% of high and very high
Age	≤40 years (median)	4.4 (0.6)	3.6 (0.7)	70.6
	41 years+	4.3 (0.6)	3.6 (0.7)	71.8
	*P* value	0.008*	0.489*	0.359***
Sex	Male	4.4 (0.6)	3.7 (0.6)	64.0
	Female	4.3 (0.6)	3.6 (0.7)	73.3
	P value	0.583*	0.117*	0.003***
Seniority in the profession	≤14 years (median)	4.4 (0.6)	3.5 (0.7)	69.4
	15 years+	4.3 (0.6)	3.7 (0.6)	72.7
	P value	0.103*	0.004*	0.138**
Country of birth	Israel	4.4 (0.6)	3.6 (0.7)	70.9
	FSU	4.3 (0.7)	3.6 (0.6)	71.4
	Other	4.3 (0.6)	3.6 (0.5)	72.6
	P value	0.044**	0.597**	0.954***
Education	RN	4.1 (0.6)	3.6 (0.6)	67.1
	RN BA	4.4 (0.6)	3.5 (0.7)	69.9
	RN MA	4.5 (0.6)	3.7 (0.6)	78.1
	P value	< 0.0001**	0.001**	0.028***
Type of Hospital	General	4.4 (0.6)	3.6 (0.6)	71.8
	Pediatric	4.2 (0.6)	3.4 (0.7)	69.3
	Psychiatric	4.4 (0.6)	3.7 (0.7)	70.5
	P value	< 0.0001**	0.002**	0.787***
Form of Employment	100%	4.4 (0.6)	3.7 (0.7)	72.0
	75–90%	4.2 (0.6)	3.4 (0.6)	67.9
	25–66%	4.2 (0.7)	3.6 (0.6)	72.6
	P value	< 0.0001**	< 0.0001**	0.461***
Area of practice	Surgery	4.4 (0.6)	3.7 (0.7)	71.7
	Psychiatry/Rehabilitation	4.4 (0.7)	3.7 (0.6)	72.7
	Intensive Care Units	4.3 (0.7)	3.5 (0.7)	72.0
	Pediatric/Newborn/Premature	4.1 (0.7)	3.5 (0.7)	75.0
	Internal Medicine/Geriatrics	4.4 (0.7)	3.6 (0.6)	66.7
	Emergency Room	4.3 (0.6)	3.6 (0.7)	83.3
	Oncology	4.1 (0.5)	3.3 (0.7)	47.2
	Obstetrics & Gynecology	4.3 (0.6)	3.6 (0.7)	76.0
	Operating room	4.7 (0.6)	3.7 (0.7)	66.7
	Other	4.2 (0.5)	3.6 (0.7)	61.5
	P value	< 0.0001**	0.007**	0.041***

*p value of *t*-test

**p value of ANOVA test

***p value of *chi-square* test

**Fig. 1:**
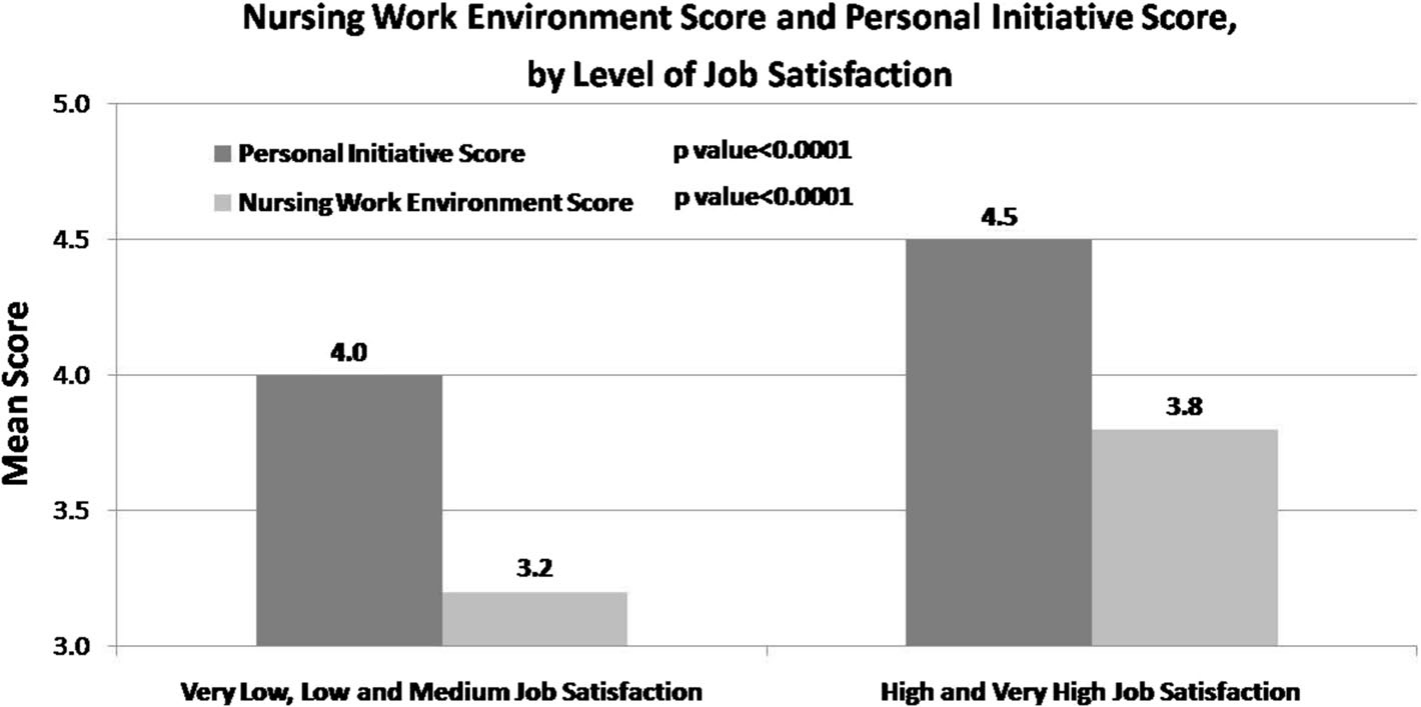
Nursing work environment and personal initiative, by job satisfaction

The Personal Initiative and Work Environment Satisfaction scores were both significantly associated with job satisfaction (Fig. 1). The mean score of personal initiative (4.5) and the mean work environment score (3.8) were significantly higher among those who reported high and very high job satisfaction compared to nurses who reported very low-medium job satisfaction (4.0 and 3.2 respectively). There was no significant interaction between personal iterative and work environment variables in relation to job satisfaction.

### Multivariable analysis of job satisfaction

Following the result of univariate analysis, a multivariable logistic regression model was used to study the association between work environment and job satisfaction (Table 3).

**Table 3:** The multivariate logistic regression final model for high and very high job satisfaction prediction

Variables	Odds Ratio (OR) [95% Confidence Interval]		
	Model I^a^	Model II^b^	Model III^c^
**Sex** female vs. male	**1.506** **[1.096–2.069]**	**1.543** **[1.105–2.155]**	**1.848** **[1.289–2.651]**
**Education** (vs. RN)			
RN BA	1.150 [0.809–1.638]	0.940 [0.651–1.358]	1.164 [0.785–1.7326]
RN MA	**1.793** **[1.145–2.808]**	1.240 [0.774–1.987]	1.290 [0.778–2.138]
**Area of practice** (vs. Oncology)			
Surgery	**2.422** **[1.201–4.884]**	1.950 [0.941–4.041]	1.524 [0.686–3.382]
Psychiatry/Rehabilitation	**2.666** **[1.320–5.386]**	**2.208** **[1.064–4.582]**	1.180 [0.495–2.814]
Intensive Care Units	**2.341** **[1.125–4.870]**	**2.165** **[1.011–4.637]**	2.058 [0.896–4.727]
Pediatric/Newborn/Premature	**2.636** **[1.209–5.748]**	**3.065** **[1.360–6.909]**	**2.845** **[1.171–6.912]**
Internal Medicine/Geriatrics	1.925 [0.897–4.130]	1.559 [0.703–3.460]	1.180 [0.495–2.814]
Emergency Room	**5.694** **[1.922–16.868]**	**5.519** **[1.794–16.973]**	**4.965** **[1.517–16.250]**
Obstetrics & Gynecology	2.968 [0.961–9.165]	2.756 [0.850–8.937]	1.976 [0.555–7.043]
Operating room	1.951 [0.636–5.978]	1.199 [0.376–3.822]	0.825 [0.226–3.017]
Other	1.839 [0.728–4.647]	1.732 [0.649–4.620]	1.424 [0.498–4.151]
**Personal Initiative Score**	**–**	**2.962** **[2.316–3.787]**	**2.817** **[2.163–3.669]**
**Work Environment Score**	**–**	**–**	**4.305** **[3.308–5.602]**

^**a**^ Model I includes sex, education, and area of practice

^**b**^ Model II includes sex, education, area of practice, and personal initiative score

^**c**^ Model III includes sex, education, area of practice, personal initiative score and nursing work environment score

In the first step, gender, education, and area of practice were included in the model of job satisfaction prediction (Table 3, Model I). Women had significantly higher odds (by 50%) for high and very high job satisfaction in comparison to men (OR = 1.506; 95% CI 1.096–2.069). The probability of nurses with a master’s degree reporting high and very high job satisfaction was almost twice as high as those with a RN certificate (OR = 1.793; 95% CI 1.145–2.808). Compared to nurses who work in oncology (reference group), nurses working in areas of practice such as surgery, psychiatry/rehabilitation, ICU, and pediatrics had significantly more than twice the chance of reporting high and very high job satisfaction. This increased to almost six-fold higher odds for high and very high job satisfaction among nurses working in the ER. This model explained only 4% of variation in job satisfaction.

After including the personal initiative score in the model (Table 3, Model II), significantly higher odds for high and very high job satisfaction were observed among women (vs. men) and education was no longer associated with job satisfaction. The association between most of areas of practice and job satisfaction found in Model I remained significant.

Every elevation of one point of the mean score for personal initiative score was associated with a significant, three-fold elevation in the odds of reporting high and very high job satisfaction (OR = 2.962; 95% CI 2.316–3.787). This model explained 15% of the variation in job satisfaction.

After including the nursing work environment score (Table 3, Model III), female gender continued to maintain a significant association with high and very high job satisfaction, while education was not associated with job satisfaction. Work in the ER and pediatric area of practice was significantly associated with five-fold and three-fold higher odds for high and very high job satisfaction in comparison to work in oncology (OR = 4.965; 95% CI 1.517–16.250 and OR = 2.845; 95% CI 1.171–6.912, respectively). The association between mean score for work environment and job satisfaction was strong and significant: every elevation of one point of the mean score for nurse work environment score was associated with a significant, four-fold higher elevation in the odds of reporting high and very high job satisfaction (OR = 4.305; 95% CI 3.308–5.602). An increase of one point in personal initiative score was associated with an almost three-fold increase in the odds for high and very high job satisfaction (OR = 2.817; 95% CI 2.163–3.669). This model explained 32% of the variation in job satisfaction.

## Discussion

The present study examined the relationships between job satisfaction, personal initiative and work environment characteristics, among nurses working in hospitals in Israel. The results indicated that higher satisfaction with the work environment and a higher personal initiative score are independently associated with higher job satisfaction when other nurses’ characteristics were taken into account. These findings support the two-factor theory of Herzberg, which claims that intrinsic and extrinsic factors are interdependent. On the other hand, according to Herzberg’s theory, extrinsic factors do not contribute to the satisfaction of the employee, but can only prevent a worker’s dissatisfaction. In our study, both work environment and personal initiative contributed to higher job satisfaction, while the work environment score provided the most significant contribution to the variation in job satisfaction. This finding may be explained by the fact that the components of the work environment assessed by the questionnaire, were nurse autonomy, control over nursing practice, nurse-physician relations, and team work environment. Thus, these components represent predominantly intrinsic rather than extrinsic factors such as work conditions, salary, status etc. The findings of our study are consistent with the meta-analysis of 31 studies on job satisfaction in nurses, which reported that lower stress in the work environment, high levels of collaboration between nurses and physicians and nurse’s autonomy, are crucial for high job satisfaction [24].

Our study suggests a possible partial mediating effect of personal initiative on the association between work environment and job satisfaction. That is, the work environment may contribute to initiative behavior, which in turn is associated with higher job satisfaction. Previous research into the factors influencing nursing leadership and personal initiative, reported that a work climate that promotes initiative is a significant factor affecting actual innovative behavior at work [19]. Personal initiative is highly dependent not only on the personality trait of the nurse, but also on the culture at the workplace. Individual workplaces may have a certain organizational culture, characteristics, resource allocations, and structures, which influence the learning environments [25]. A supportive environment that promotes learning and innovation, encourages personal growth and contributes to higher job satisfaction partially through personal initiative.

Regarding the differences between wards, specific clinical departments (pediatric and ER) were directly associated with higher job satisfaction, independently from other nurses` characteristics and independently from work environment and personal initiative scores. When the oncology department served as a reference group (the odds for high and very high job satisfaction were lowest in the oncology area), working in the ER and pediatric area were associated with the highest odds for high and very high job satisfaction. These findings can be explained by different exposure to traumatic experiences and emotional burden among these nurses. Oncology nurses consistently encounter high mortality rates among their patients and are consistently exposed to a variety of stressors, which may explain the high stress and burnout seen in this specialty [26, 27]. Our findings of the highest odds for high and very high job satisfaction in the ER are consistent with some previous studies but contradict another, although the comparison between studies is problematic due to the use of different tools for measuring job satisfaction. A literature review of the differences in job satisfaction by ward revealed limited and inconsistent findings. A study from Greece reported similar job satisfaction levels among nurses from the ICU and ER [28]. However, a study from Brazil reported higher job satisfaction in the ER than in the ICU [29]. Employees in pediatric units were the most satisfied, according to a study conducted in the US [30], and nurses working in medical/surgical units were the most satisfied according to another [31]. In the current study, working in the ER and in a pediatric ward were associated with higher job satisfaction even when personal initiative and work environment were taken into account. Given the differences in work patterns, with chronic versus acute conditions, the motivational aspects of job satisfaction may play a significant role. In a ward treating patients in acute conditions, the immediate effect of nursing intervention faster influence may have a more positive impact on job satisfaction.

In the current study, higher education was significantly associated with higher job satisfaction, but this effect disappeared once personal initiative was taken into account. A previous study reported that educational interventions may contribute significantly to developing leadership behaviors among nurses [32]. This may explain our findings of the mediating effect of personal initiative as represented by the disappearance of the association between education and job satisfaction after the inclusion of personal initiative in the model.

Finally, women in our study had higher odds for high and very high job satisfaction than men. This finding is consistent with most of the previous studies that explored the association between gender and job satisfaction among nurses, and reported that women demonstrated higher job satisfaction [33–35]. One possible explanation for this finding is that men are more influenced by extrinsic rewards such as salary [35]. Another explanation may be related to the findings that male nurses experience more aggression [36], and more discrimination in the workforce [37]. They may also be frustrated by the role strain of being a male in a female-dominated profession [35, 38], and perceive their social status as “taking a step down” [35].

### Limitations

Due to the cross-sectional design, we cannot draw conclusions about the causality of the associations found in the study, but only about the association between the variables. Another limitation is that the assessment of extrinsic factors of the work environment such as payment, workload, staffing levels and professional status was not collected in this study. As the final model explained almost a third of variance, additional characteristics of participants and work environment that were not assessed in this study may also contribute to job satisfaction.

## Conclusions and implications

The unique contribution of this study relates to the incorporation of the personal initiative characteristic into the model of relationships between work environment and job satisfaction among nurses. The findings of the current study demonstrate that high personal initiative, together with a positive perception of the nursing work environment, contribute significantly to the explanation of job satisfaction among young nurses with an advanced academic background.

The ability to initiate is a crucial element in promoting innovations. The significant associations between job satisfaction, the personal initiative and work environment, emphasize a need to invest more efforts in encouraging nurses, especially those from the new generation, to be active, share knowledge, and promote innovation. On a personal level, it is important to “provide space” and to encourage nurses to initiate. Additionally, on the departmental levels, the nursing leadership need to improve the work environment, especially with regard to nurses’ autonomy, their professional status, and the teamwork milieu. The findings of this study emphasize the need to “tailor a personal suit” for different intervention programs according to the type of clinical ward. In further research, we recommend the examination of additional variables such as leadership, managerial support, and encouragement to initiate that may influence the relationships between personal initiative and job satisfaction.

## Data Availability

The data that support the findings of this study are available from the corresponding author upon reasonable request.

## Abbreviations

FSU: Former Soviet Union
ICU: Intensive Care Unit
OR: Operation Room
ER: Emergency Room
RN: Registered Nurse
RN BA: Registered nurse (RN) with Bachelor’s degree (BA)
RN MA: Registered nurse (RN) with Masters’s degree (MA)
OR (statistics): Odds Ratio
SD: Standard Deviation
CI: Confidence Interval
